# Two new species of betatorqueviruses identified in a human melanoma that metastasized to the brain

**DOI:** 10.18632/oncotarget.22400

**Published:** 2017-11-11

**Authors:** Terry Fei Fan Ng, Jennifer A. Dill, Alvin C. Camus, Eric Delwart, Erwin G. Van Meir

**Affiliations:** ^1^ Blood Systems Research Institute, San Francisco, California, USA; ^2^ Department of Laboratory Medicine, University of California at San Francisco, San Francisco, California, USA; ^3^ Department of Pathology, University of Georgia, Athens, Georgia, USA; ^4^ Departments of Neurosurgery and Hematology & Medical Oncology, Winship Cancer Institute and School of Medicine, Emory University, Atlanta, Georgia, USA; ^5^ Current/Present address: DVD, NCIRD, Centers for Disease Control and Prevention, Atlanta, Georgia, USA

**Keywords:** brain tumor, neuro-oncology, anellovirus, metagenomics, metastasis

## Abstract

The role of viral infections in the etiology of brain cancer remains uncertain. Prior studies mostly focused on transcriptome or viral DNA integrated in tumor cells. To investigate for the presence of viral particles, we performed metagenomics sequencing on viral capsid-protected nucleic acids from 12 primary and 8 metastatic human brain tumors. One brain tumor metastasized from a skin melanoma harbored two new human anellovirus species, Torque teno mini virus Emory1 (TTMV Emory1) and Emory2 (TTMV Emory2), while the remaining 19 samples did not reveal any exogenous viral sequences. Their genomes share 63-67% identity with other TTMVs, and phylogenetic clustering supports their classification within the *Betatorquevirus* genus. This is the first identification of betatorqueviruses in brain tumors. The viral DNA was in its expected non-integrated circular form, and it is unclear if the viruses contributed to tumor formation. Whether the viruses originated from blood, or the primary skin tumor could not be ascertained. Overall, our results demonstrate the usefulness of viral metagenomics to detect previously unknown exogenous virus in human brain tumors. They further suggest that active viral infections are rare events in brain tumors, but support a follow-up larger scale study to quantify their frequency in different brain tumor subtypes.

## INTRODUCTION

Cancers largely result from genetic mutations that induce cell transformation. While the role of some viruses in animal carcinogenesis is well established, virus-mediated oncogenesis has only been shown for a small number of human cancers. These oncoviruses include human alpha papillomaviruses (mainly HPV16 and 18), Kaposi's sarcoma-associated herpesvirus (HHV8), Merkel cell polyomavirus, Epstein–Barr virus, Human T-lymphotropic virus type 1, and hepatitis B virus [1–3]. Human immunodeficiency virus (HIV) and hepatitis C virus (HCV) may cause cancer by more indirect mechanisms such as chronic inflammation and immune-deficiency. Genetically engineered oncolytic viruses are also being developed as therapies against brain tumors [4–6].

What triggers the genetic mutations that cause primary brain tumors is mostly unknown. In healthy individuals, the brain is considered a privileged organ where the blood-brain barrier prevents pathogens from entry [7], but when pathogens, including viruses, gain access by crossing the blood-brain barrier, it can lead to diseases such as encephalitis or microcephaly in developing fetuses or infants [8, 9]. Currently, the only known virus capable of causing a brain tumor in a mammalian host is a raccoon polyomavirus [10]. No virus has definitively been demonstrated to cause primary brain tumors in human, yet a variety of viruses have been reported to be associated with primary human brain tumors, including polyomaviruses and cytomegaloviruses [11, 12].

**Figure 1:**
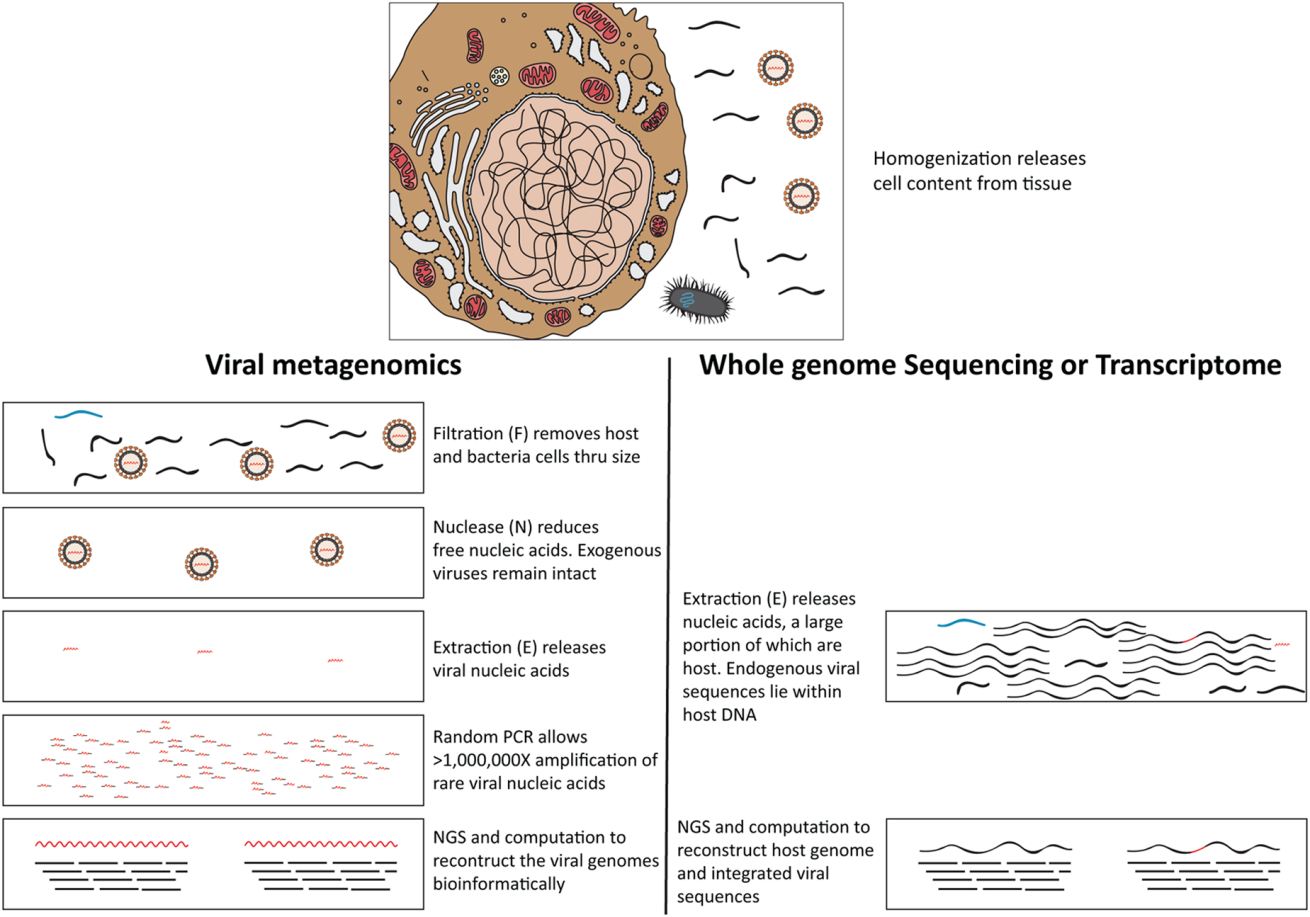
Schematic diagram outlines the typical viral metagenomic approach in this study, using filtration, nuclease and extraction (FNE) treatments [16, 17] to distinguish rare viral sequences from abundant host cell and free DNA Black wavy lines denote host nucleic acids; blue wavy lines denote bacterial nucleic acids; red wavy lines denote viral nucleic acids. In the top panel, host (Left), bacteria (bottom), and viruses (right) are schematically represented. Viral nucleic acids are protected by viral capsids from degradation during nuclease (N) treatments, unlike the host and bacterial nucleic acids. Obtaining rare exogenous viral sequence through viral metagenomics (bottom panel left) is different from obtaining endogenous viral genomes through transcriptome or whole genome sequencing (bottom panel right).

Due to their location and therapeutic resistance [13], brain neoplasms are among the most challenging to treat, and biopsies can only be obtained when medically necessary. Most knowledge of virus infections in the central nervous system comes from the analysis of cerebrospinal fluid (CSF) [7]. Analyzing brain tumor tissue directly to characterize its virome is an approach rendered possible by the recent development of next generation sequencing (NGS) methods. For example, the NGS approach allowed diagnosis of a rare leptospira infection in a case of unexplained acute meningoencephalitis in a clinically relevant timeframe [14]. A recent whole genome and transcriptome study directly sequenced the DNA and RNA from 1,122 adult diffuse gliomas, but did not report any viral sequences in the glioma and associated stroma cell genomes [15]. Whole genome or transcriptome approaches focus on host genomes or endogenous/integrated viral sequences, but are less sensitive for detecting encapsidated viral genomes (Figure 1).

To address this issue, we applied a viral metagenomics approach to focus sequencing on nuclease-resistant (encapsidated) viral nucleic acids after filtration of tissue homogenates through a 400nm filter [16, 17] (Figure 1), to investigate 20 retrospectively collected frozen human brain tumors of various types.

## RESULTS AND DISCUSSION

**Figure 2:**
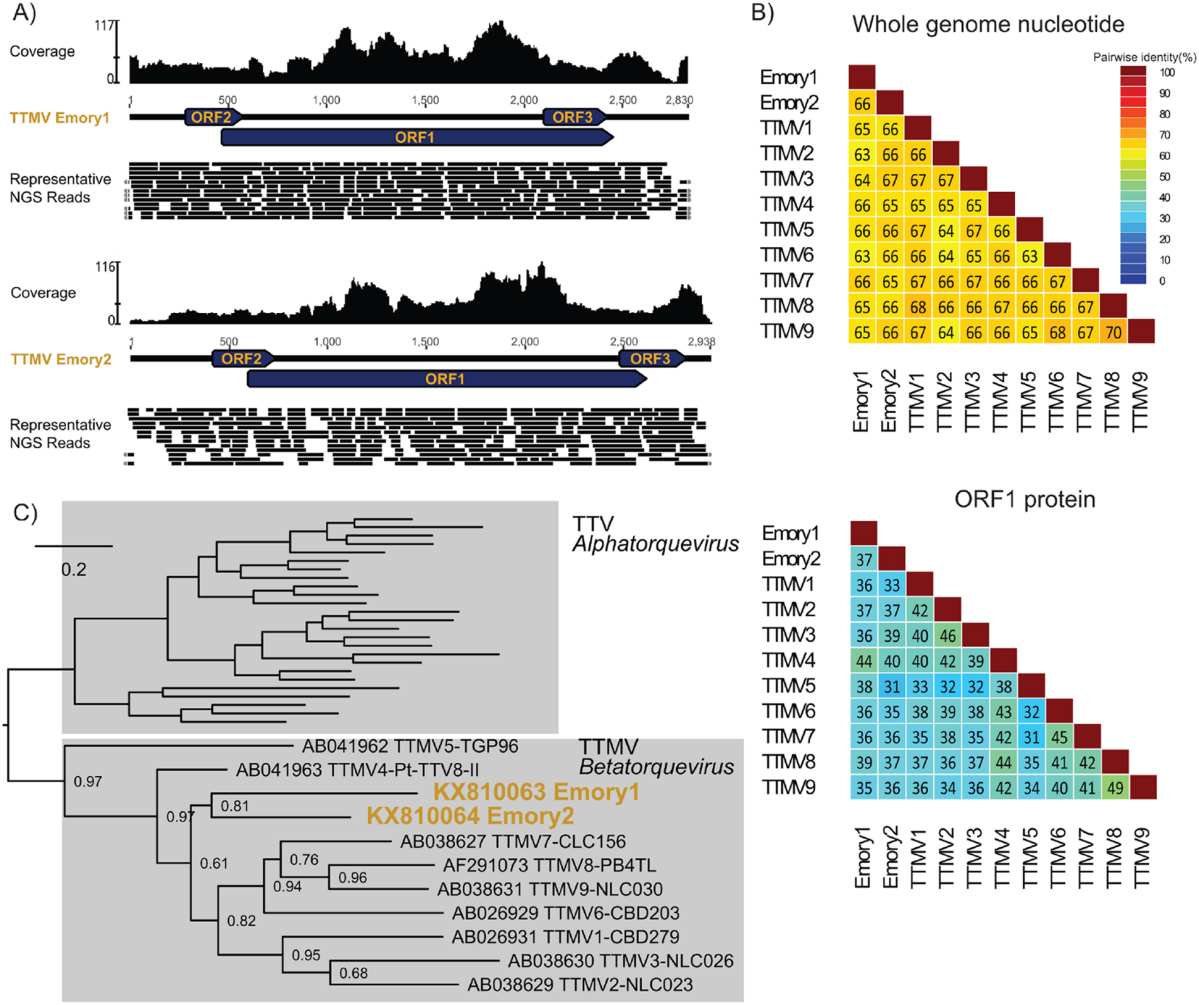
Comprehensive genomic and phylogenetic analysis of the Torque teno mini virus Emory1 (TTMV Emory1) and Torque teno mini virus Emory2 (TTMV Emory2) **(A)** Schematic depiction of the TTMV Emory1 and Emory2 genome organization. Coverage for NGS data is indicated, and a portion of the representative NGS reads is shown. The circular genome is graphically linearized for display purpose, and the reads covering across the ends are marked with grey arrows on the extremities. **(B)** Pairwise whole genome nucleotide identities and ORF1 amino acid identities between Torque teno mini viruses. Protein sequences were aligned with MUSLE [52], and sequence identities were calculated using the species demarcation tool [53]. **(C)** ORF1 protein phylogeny of the genus *Betatorquevirus*, including the newly described TTMV Emory1 and Emory2. Maximum likelihood phylogeny was generated with PhyML [54], where branch support was calculated using Approximate Likelihood-Ratio Test (aLRT). The scale bar represents evolutionary distance in substitutions per site.

**Figure 3:**
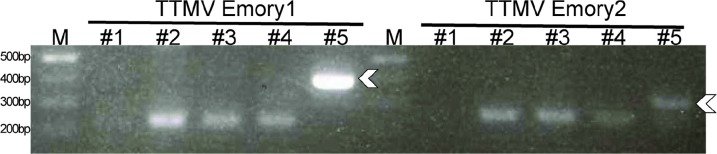
PCR analysis of viral DNA Arrows denote expected amplicon. TTMV Emory 1 PCR was performed with primers Emory1_AF and Emory1_AR with an amplicon size of 387 bp. TTMV Emory 2 PCR was performed with primers Emory2_AF and Emory2_AR with an amplicon size of 332 bp. M, size markers; #1-5, samples 1-5. Bands below the expected amplicon size were due to primers or primer dimers.

The virome and PCR analyses identified two novel anelloviruses in a specimen from a patient with a skin melanoma that had metastasized to the brain collected in 1993 (Sample #5) (Figures 2 and 3). Genome assembly from sequenced fragments evidenced circular viral genomes and these did not originate from cancer cell chromosomal DNA, as no contiguous viral-host genome fragments were found (Figure 2A). No viral sequences were detected in the other tumors. In-silico investigation of the entire in-house viral metagenomic database [18] of the laboratory where the NGS was performed did not detect any other samples containing sequences of these two viruses, ruling against possible lab contamination. Anelloviruses in the family *Anelloviridae* comprise small, non-enveloped viruses containing a single-stranded, negative-sense circular DNA genome enclosed within an icosahedral nucleocapsid. The genome of anelloviruses ranges between 2.1–3.9 kb in length. Human infections with anelloviruses are virtually ubiquitous [19–21], and vertebrate homologs have been described in various domestic animal species including dogs, cats, pigs, cows, chickens, and sheep, as well as wild non-human primates and marine mammals [22–27]. There are three known anellovirus genera that infect humans (*Alphatorquevirus, Betatorquevirus* and *Gammatorquevirus*). The *Alphatorquevirus* genus includes 29 species (*Torque teno virus, TTV 1–29*), *Betatorquevirus* includes 12 species (*Torque teno mini virus, TTMV 1–12*), and *Gammatorquevirus* includes 15 species (*Torque teno midi virus, TTMDV 1–15*) [28]. Human anelloviruses are commonly found in human blood, but can also be detected in liver, kidney, lungs, spleen, and occasionally in cerebrospinal fluid [29–31], brain tissue [30], and nerve tissue [31]. The detection of anellovirus in brain is rare; the literature mentions brain tissue from a single adult who suffered from head trauma tested positive for an Alphatorquevirus [30]. Since their discovery, human anelloviruses association with various diseases has been proposed, including hepatitis and respiratory illnesses, but strong and consistent evidence for disease association and pathogenesis in humans is lacking [20, 32].

Phylogenetic analysis of the ORF1 gene, the most conserved open reading frame (ORF) among anelloviruses, showed that the two viruses clustered with other Torque teno mini viruses (Figure 2C), supporting their phylogeny in the *Betatorquevirus* genus. They were designated Torque teno mini virus Emory1 (TTMV Emory1, KX810063) and Torque teno mini virus Emory2 (TTMV Emory2, KX810064), and their genomes were deposited in GenBank. The genome lengths for TTMV Emory1 and Emory2 are 2,830 and 2,938 nucleotides (nt), with a GC content of 37% and 36%, respectively. Both viral genomes contain the typical anelloviruses genome organization [28] (Figure 2A), with three open reading frames where ORF1 is the longest. Compared to known species of TTMV and with each other, both viruses share 33-44% amino acid identity and 62-65% nucleotide identity (NI) in ORF1, as well as 63-67% NI in the whole genome (Figure 2B). Current International Committee on Taxonomy of Viruses (ICTV) criteria state that anelloviruses of the same species share at least 65% nucleotide identity in ORF1. Given their sequence divergence, TTMV Emory1 and Emory2 likely each represent new human anellovirus species within the genus *Betatorquevirus*.

This pairwise comparison, together with the phylogenetic analysis (Figure 2), strongly support that TTMV Emory1 and Emory2 are distinct taxa. The presence of two distinct anellovirus genomes in a single brain tumor supports co-infection with two anelloviruses.

In an attempt to localize the viruses within a specific cell type within the tumors, we performed *in situ* hybridization (ISH) and fluorescence *in situ* hybridization (FISH) on frozen tumor sections post-fixed with formalin with probes specific to Emory1 and Emory2. However, results for both methods were negative (data not shown). As a control, DNA of both viruses was confirmed present by PCR in the pre-fixation frozen specimens (Figure 3). This suggests that the viral nucleic acid in tumor tissues was likely beyond the limits of detection of *in situ* hybridization, or that anelloviruses were present as viral particles in blood circulating through the tumor, which would have been lost during the fixation steps used for sample preparation. We also attempted to obtain the primary skin melanoma tumor for virome analysis, but the original samples were no longer available.

The most common intracranial neoplasms in adults originate from systemic tumors that metastasize to the brain. The most frequent are lung carcinomas (∼20%), melanomas (∼7%), renal cell carcinomas (∼6.5%), and breast carcinomas (∼5%) [33]. In this study, we investigated a variety of brain tumor types (Table 1). We only found a single tumor out of 20 to contain exogenous viral sequences (rather than human endogenous retroviruses). It is, therefore, unlikely that viruses are commonly replicating at high levels in these tumors.

**Table 1:** Human brain tumor specimens used for the study

Sample number	Description	Age/sex	Weight
1	Metastasis, Carcinoma, primary of breast origin	49/F	233 mg
2	Metastasis, Carcinoma, poorly differentiated, primary of unknown origin	67/M	270 mg
3	Metastasis, Carcinoma, poorly differentiated, primary of unknown origin	40/F	351 mg
4	Metastasis, Adenocarcinoma with lung primary	49/F	291 mg
5	Metastasis, Melanoma, malignant, primary from skin	57/M	250 mg
6	Metastasis, Melanoma, malignant, primary from skin	51/M	244 mg
7	Metastasis, Adenocarcinoma, primary of breast origin	38/F	224 mg
8	Metastasis, Carcinoma, poorly differentiated, consistent with breast primary	67/F	244 mg
9	Schwannoma (I)	32/F	264 mg
10	Schwannoma (I)	49/M	223 mg
11	Medulloblastoma (IV)	7/M	231 mg
12	Medulloblastoma (IV)	34/F	213 mg
13	Pilocytic Astrocytoma (I)	8/F	237 mg
14	Anaplastic Astrocytoma (III), recurrence from pilocytic astrocytoma (I)	76/M	225 mg
15	Glioblastoma (IV), recurrence from oligodendroglioma (II)	44/F	251 mg
16	Glioblastoma (IV)	55/M	257 mg
17	Gliosarcoma (IV)	59/M	222 mg
18	Gliosarcoma (IV)	83/F	210 mg
19	Subependymoma (I)	77/M	263 mg
20	Subependymoma (I)	59/M	243 mg

WHO 2006 classification grade is indicated for primary brain tumors. Weight of frozen sample used for analysis is indicated.

Our study did not identify polyomaviruses or cytomegalovirus sequences in the filtered and nuclease treated tumor homogenates, even though these viruses were previously reported in primary human brain tumors [11, 12]. This could be due to limited sampling, or because these hypothetical oncogenic viruses are integrated into the tumor genome after initial infection [34] – thus yielding no viral particles. The latter explanation is unlikely, as efforts aimed at detecting viral sequences in human cancer genomes confirmed human papillomavirus, hepatitis B virus and Epstein-Barr virus transcripts in carcinomas affecting the cervix, liver, or lymphocytes, but failed to identify viral sequences in human brain tumors and skin melanomas [35]. Another virome assessment using glioblastoma datasets from the Cancer Genome Atlas (TCGA) did not find anelloviruses in the tumor by nucleotide homology search [36]; however, the search database was limited to less than 40 anellovirus genomes.

Our study does not provide any evidence that the newly discovered viruses had a role in the pathogenesis of the metastatic melanoma in which they were identified. Although anelloviruses nearly universally infect humans in their lifetime, no human anellovirus has yet been shown to be oncogenic [20]. Genomes of the *Anelloviridae* family lack homologs of genes that promote cell transformation in other DNA viruses such as polyomaviruses or papillomaviruses, which respectively contain oncogenic proteins large T antigen and E6 and E7 that inactivate the retinoblastoma (Rb) and p53 tumor suppressors. Anelloviruses are not known to integrate into host cell genomes [37], thereby excluding insertional mutagenesis. Some studies have suggested TTMVs may have indirect carcinogenic effects by modulating T cell immune responses, possibly through expression of specific miRNAs that inhibit the interferon response [38, 39]. If future larger scale studies confirm their presence in specific cancers, then further studies on potential oncogenic effects will be warranted.

Tumors undergo extensive angiogenesis, increasing the number of blood vessels within the neoplastic tissue [40]. The disorganized nature of the vasculature can result in increased vascular permeability and disruption of the blood-brain-barrier [41, 42], rendering the neoplastic tissue more susceptible to the extravasation of viruses from the blood, such as anelloviruses. Several studies have detected anelloviruses in cerebrospinal fluid, which directly bathes the brain [29–31]. Immunosuppression during organ transplant, HIV/AIDS, and sepsis can also cause increases in anellovirus titers circulating in the blood [43–46]. Whether anelloviruses can infect some metastatic brain tumor cells or their detection here simply reflects viremia and blood present in cancer biopsies remains to be determined.

In conclusion, our study identified two new species of betatorqueviruses and is the first demonstration of TTMVs in human brain tumor specimens. Furthermore, it provides proof-of-principle that metagenomic analysis can be used to identify viral genomes that are not integrated in human brain tumor genomes, likely reflecting their presence as infectious viral particles. Finally, it suggests that active virus infections are rare in human brain tumors, complementing prior studies that focused only on transcriptome or viral DNA integrated in tumor cells.

## MATERIALS AND METHODS

### Sample collection

We investigated 20 frozen human brain tumor samples retrospectively collected under institutional IRB approval, including primary tumors of Schwannoma, Medulloblastoma, Astrocytoma, Glioblastoma, Gliosarcoma, and Subependymoma, as well as metastases to brain from Carcinoma, Melanoma, and Adenocarcinoma (Table 1; WHO 2006 classification). The tumor specimens were collected from neurosurgeries under IRB approval, and were stored in liquid nitrogen vapor at −130°C. The samples were distributed into four pools, each containing five tumor samples (specimens #1-5; 6-10; 11-15 and 16-20) for viral metagenomics (Figure 1).

### Viral metagenomic sequencing

Unbiased viral metagenomics sequencing of nuclease-resistant nucleic acids was performed directly according to previously described protocols that detect both DNA and RNA viruses [16, 17]. Briefly, homogenized tumor pools were filtered through 400 nm filters (Millipore), followed by depletion of host nucleic acids in the filtrate using DNAse and RNAse. Nuclease-resistant nucleic acids were extracted using the QIAamp Viral RNA Mini Kit (Qiagen; which extracts both DNA and RNA [47]) and sequence-independent amplification was performed using random priming. Random first strand synthesis of both DNA and cDNA was performed using Superscript IV (Invitrogen; which has both reverse transcriptase and DNA polymerase activities) and primer N1_8N 5′-CCTTGAAGGCGGACTGTGAGNNNNNNNN-3′. The second strand was synthesized using Klenow fragment DNA polymerase (New England BioLabs) using the same primer. The products from the second strand synthesis were then used as input to be randomly amplified by PCR using AmpliTaq Gold DNA polymerase and primer N1, 5′-CCTTGAAGGCGGACTGTGAG-3′. Amplified DNA were subjected to Nextera XT DNA Sample Prep kit, and sequenced using the MiSeq (Illumina) sequencing system with 2 × 250 bp paired-end sequencing reagents [16]. This method has been used extensively for both DNA and RNA exogenous viruses [16, 17, 47].

### Bioinformatics analysis

Sequence data was analyzed using a customized NGS pipeline as described previously [48]. First, reads identical to human and bacterial genomes were computationally subtracted [16, 17]. The remaining reads were assembled *de novo* into contigs using Ensemble Assembler [48]. The contigs and the unassembled reads were then aligned to an in-house viral proteome database using BLASTx with an E-value cutoff of 0.01. The significant sequence matches to virus were filtered again with an in-house non-virus-non-redundant (NVNR) universal proteome database using BLASTx.

Genome analysis was performed according to previously described procedures [49], including genome organization, ORF annotation, NGS coverage analysis, pairwise comparison of the ORF1 gene and whole genomes, and phylogenetic analysis. Viral sequences identified in the virome analysis were reconfirmed in the specific individual samples using PCR (Figure 3).

### PCR and *in situ* hybridization

Specific PCR primers for each new TTMV were designed directly from the genomes obtained from the NGS data. Tumor DNA was extracted using QIAamp Viral RNA Mini kit (Qiagen). PCR was performed using primer sets specific for each virus: Emory1_AF, 5′-CGCCGAAAACCTTACAAAAA-3′; Emory1_AR, 5′-TTGGTGGTTGTGTGCTGAAT-3′; Emory2_AF, 5′-CCACCACAACAATTCCAAAA-3′; Emory2_AR, 5′-CAGTCTCCGCTCATTGGTTT-3′. PCR was carried out using the Ex Taq DNA Polymerase (Clontech) for 45 cycles using a touch down cycling condition as described before [50]: 95°C for 5 min, 45 cycles of [94°C for 1 min, 58°C minus 0.2°C per cycle for 1 min, 72°C for 3 min], followed by 72°C for 10 min.

*In situ* hybridization (ISH) and fluorescence *in situ* hybridization (FISH) were performed on frozen tumor sections post-fixated with formalin according to an established protocol [51]. Digoxigenin-labeled PCR probes were generated using primers specific to Emory1 and Emory2; the primers were Emory1_AF, Emory1_AR, Emory2_IF, 5′-CCAACGACCTCGAAAACATT-3′; Emory2_IR, 5′-AGTTGTCGGAGCTGCTGTTT-3′. Sections of formalin-fixed tissue were rehydrated, digested with proteinase K and then the probes were applied to glass slides and allowed to hybridize overnight in a 37°C humidified oven. After washing, sections were blocked with universal blocking buffer (BioGenex). Mouse anti-digoxigenin antibody (ROCHE) diluted 1:500 with Antibody Diluent (DAKO) was applied to the sections for 60 minutes and then washed. Detection of bound antibody was done by serial application of goat anti-mouse biotinylated immunoglobulins (Biogenex), streptavidin alkaline phosphatase (Biogenex), and napthol fast red substrate (DAKO), followed by mounting with aqueous adhesive. For FISH, sections were blocked with normal goat serum (Rockland), conjugated with Streptavidin-Alexa Fluor^®^ 532, and mounted with ProLong Gold with DAPI (Molecular Probes).
